# Decreased Tactile Sensitivity Induced by Disownership: An Observational Study Utilizing the Rubber Hand Illusion

**DOI:** 10.3389/fnsys.2021.802148

**Published:** 2022-01-20

**Authors:** Kota Ataka, Tamami Sudo, Ryoji Otaki, Eizaburo Suzuki, Shin-Ichi Izumi

**Affiliations:** ^1^Department of Physical Medicine and Rehabilitation, Tohoku University Graduate School of Medicine, Sendai, Japan; ^2^Department of Rehabilitation, Tohoku University Hospital, Sendai, Japan; ^3^Department of Computer and Information Sciences, Graduate School of Engineering, Tokyo University of Agriculture and Technology, Tokyo, Japan; ^4^Department of Rehabilitation, Yamagata Saisei Hospital, Yamagata, Japan; ^5^Department of Physical Therapy, Yamagata Prefectural University of Health Sciences, Yamagata, Japan; ^6^Department of Physical Medicine and Rehabilitation, Tohoku University Graduate School of Biomedical Engineering, Sendai, Japan

**Keywords:** body ownership, disownership, multisensory integration, rubber hand illusion, tactile sensitivity, sensory impairment, rehabilitation

## Abstract

The sense of body ownership, the feeling that one’s own body belongs to oneself, is generated from the integration of visual, tactile, and proprioceptive information. However, long-term non-use of parts of the body due to physical dysfunction caused by trauma or illness may disturb multisensory integration, resulting in a decreased sense of body ownership. The rubber hand illusion (RHI) is an experimental method of manipulating the sense of ownership (SoO). In this illusion, subjects feel as if the rubber hand in front of them were their own hand. The RHI elicits the disownership phenomenon; not only does the rubber hand feels like one’s own hand, but one’s own hand does not feel like one’s own hand. The decrease of ownership of one’s own body induced by the bodily illusion is accompanied by neurophysiological changes, such as attenuation of somatosensory evoked potential and decreases in skin temperature. If the loss of the SoO is associated with decreased neurophysiological function, the dysfunction of patients complaining of the loss of ownership can be exacerbated; appropriate rehabilitation prescriptions are urgently required. The present study attempted to induce a sense of disownership of subjects’ own hands using the RHI and investigated whether the tactile sensitivity threshold was altered by disownership. *Via* questionnaire, subjects reported a decrease of ownership after the RHI manipulation; at the same time, tactile sensitivity thresholds were shown to increase in tactile evaluation using the Semmes-Weinstein monofilaments test. The tactile detection rate changes before and after the RHI were negatively correlated with the disownership-score changes. These results show that subjects’ sense of disownership, that their own hands did not belong to them, led to decreases in tactile sensitivity. The study findings also suggest that manipulating of illusory ownership can be a tool for estimating the degree of exacerbation of sensory impairment in patients. Consideration of new interventions that optimize the sense of body ownership may contribute to new rehabilitation strategies for post-stroke sensory impairment.

## Introduction

Sense of ownership (SoO) is the feeling that parts of the body, or the entire body, belong to oneself (Gallagher, 2000). This subjective experience is generated from multisensory integration of visual, proprioceptive, and somatosensory information through comparisons between the visually perceived body and the anatomical model of the bodily self (Gallagher, 2000; Jeannerod, 2003; Ehrsson et al., 2004; Tsakiris, 2010). When one’s own hand is observed in the appropriate position, as part of one’s body, and can be moved according to one’s own will, the hand can be clearly recognized as part of one’s own body. This conscious experience is crucial to the proper perception of information from the surroundings and to the corresponding adaptive movement.

The sense of body ownership can be altered selectively by stroke-induced brain damage, interfering with multisensory integration. Patients’ complaints like that I feel that this hand that I am now observing is not my own are occasionally reported in clinical cases. According to an observational study of stroke patients using the Visual Analog Scale (VAS) to investigate body disownership after brain damage, body disownership was also detected in cases with no obvious agnosia. Moreover, in patients with more severe impairment of motor and sensory function, more reduction in body ownership has been detected (Ronchi et al., 2020). In more serious cases, asomatognosia (unawareness of or ignoring parts or sides of the body), and somatoparaphrenia (a syndrome that includes unawareness of ownership of body parts, delusional misidentification, and anthropomorphism) due to stroke are known to cause symptoms such as loss of body ownership and attribution of the limb to another person (Feinberg and Venneri, 2014; Romano and Maravita, 2019).

The principal sources of these complaints from stroke patients include not only the primary factor, such as damage to the areas responsible for motor and sensory function, but also secondary factors, such as decreased frequency of use. Use-dependent plasticity is the brain’s ability to adapt to various changes in the surrounding environment (Nudo and Milliken, 1996; Nudo et al., 1996a,b). When use of the body part corresponding to impairment is drastically reduced, less of the cortical area is afforded to the corresponding body part over time (Liepert et al., 1995); motor training involving frequent use of a specific part of the body increases cortical representation and improves motor function accordingly (Nudo et al., 2001). Therefore, in stroke rehabilitation it is important to provide training to increase the frequency of use of paretic limbs, such as Constraint-Induced (CI) movement therapy (Liepert et al., 2000; Taub et al., 2002). Stroke patients often learn to use their intact limbs to perform compensatory actions for paretic limbs, a phenomenon called “learned nonuse” that further reduces the frequency of use of the paretic limb (Taub et al., 2006). Learned nonuse is due primarily to somatosensory deafferentation, according to a lesion study disrupting the dorsal horn of the spinal cord corresponding to a monkey’s unilateral forelimbs (Taub, 1980). Moreover, a study examining the factors associated with the frequency of paretic limb use in stroke patients revealed that both motor and sensory functions, especially tactile sensation, determine the contribution of paretic limbs to ADL (Tashiro et al., 2021). Thus, improvement of sensory deficits in stroke rehabilitation is important to prevent reduced frequency of paretic limb use.

Psychophysical experiments using bodily illusion have been widely used in recent years to elucidate the mechanism of the occurrence of a sense of body ownership. Among the experiments utilizing bodily illusion, the rubber hand illusion (RHI), first reported by Botvinick and Cohen (1998), is the most typical paradigm. Since then, many experimental studies applying this illusion have revealed that changes in the sense of body ownership have a significant effect on motor, sensory, and physiological functions. With motor function, physiological evidence has been provided to show that the amplitude of the motor-evoked potential recorded from the real hand is significantly reduced with respect to baseline during the RHI experience (Della Gatta et al., 2016). Other studies measuring somatosensory evoked potentials (SEPs) for visuo-tactile stimulation in bodily illusions have shown that experimentally induced illusory body ownership modulates activity in the primary somatosensory cortex as well as in the temporo-parietal cortex and frontal cortex, and simultaneously attenuates somatosensory precision in order to resolve conflicting multisensory input (Dieguez et al., 2009; Aspell et al., 2012; Zeller et al., 2015). In addition, taking ownership of artificial body parts *via* RHI has been demonstrated to cause physiological changes, such as reduced skin temperature of the real hand (Moseley et al., 2008; Salomon et al., 2013), and decreased tactile detection (Zopf et al., 2011; Rossi Sebastiano et al., 2021).

A significant amount of neurological evidence has been obtained through studying illusory ownership of the virtual body. However, it remains unclear whether the attribution of ownership to the virtual body is complementary to the disownership of the actual body. In a study with a psychometric approach to the RHI, subjects reported feeling that their own hands had been replaced with rubber hands, rather than that a new third hand had been added (Longo et al., 2008). A study focusing on disownership during the RHI supported the claim that the conscious experience of disownership can be induced by illusion (Lane et al., 2017). Thus, several studies support the claim that the decrease of ownership of the actual body is caused by the attribution of ownership to the virtual body. From these findings, the manipulation of body ownership by the bodily illusion is understood to reproduce a condition similar to that of a patient who complains of decrease of body ownership due to brain damage.

In the present study, we hypothesized that the introspection on decreased body ownership in stroke patients has analogical properties to the decrease in body ownership caused by the bodily illusion. Observing changes in subjects’ ownership of their own hands during studies utilizing the RHI leads to an understanding of the relationship between the patient’s decrease of body ownership and the change of sensory function in clinical practice. To examine the effect of disownership on tactile sensation, this study investigated healthy subjects’ perceived changes in the ownership of their real hands due to the RHI as well as changes in the tactile pressure detection rate before and after the illusory manipulation. Clarifying the relationship between changes in ownership and tactile sensation can potentially contribute to the development of new rehabilitation strategies that improve sensory impairment by optimizing perceptions of body ownership of patients with post-stroke sensory impairment.

## Materials and Methods

### Participants

Twenty-six healthy adults (16 male, 25 right-handed, mean age = 29.4 ± 4.6 years) were recruited by posting notices within the Tohoku University campus and at Tohoku University Hospital and by publishing the recruitment information on the Miyagi Occupational Therapists’ Association website. Handedness was assessed by way of the Edinburgh Handedness Inventory (Oldfield, 1971). All participants self-reported being healthy with normal or normally corrected vision, normal sense of touch, and no history of neurological or psychiatric disorders. The purpose and methods of the study were fully explained to the participants in writing and orally before the study was conducted. After confirming their understanding of the study, participants consented to participate in the study willingly. This study was conducted with the approval of the Ethics Committee of Tohoku University School of Medicine (registration number: 2020-1-47).

### Procedure

**FIGURE 1:**
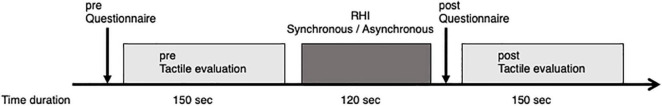
Schematic representation of the series of experimental operations. The experiment was conducted based on the A–B or B–A experimental design, composed of synchronous and asynchronous conditions. Each condition was composed of a series of operations consisting of a questionnaire, tactile evaluation, and RHI. The baseline (denoted as pre) of illusion rating questionnaires and tactile evaluation was performed prior to the start of the 120 s of visuo-tactile stimulation. Immediately following the visuo-tactile stimulation, illusion rating questionnaires and tactile evaluation was performed again (denoted as post).

The series of experiments consisted of three components: the RHI, questionnaire and tactile evaluation. Each operation was carried out in the order shown in Figure 1. The details of each operation are described below.

#### The Rubber Hand Illusion

**FIGURE 2:**
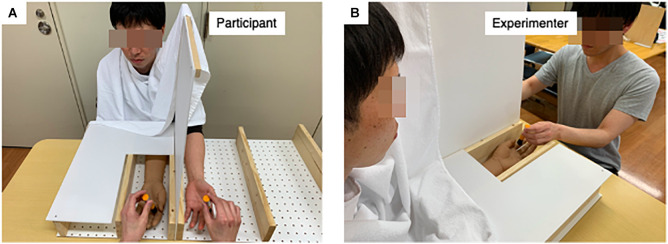
Settings of the rubber hand illusion (RHI). **(A)** Settings of the RHI from the experimenter’s viewpoint. The RHI box is a (450 mm long × 900 mm wide × 100 mm high) made of wood and plastic materials. **(B)** Presentation of the RHI stimuli from the subjects’ viewpoint.

The study’s RHI manipulation procedure was based on the method of Botvinick and Cohen (1998) with some modifications (see Figure 2). Each subject was seated in a chair with a backrest, with the chair placed in front of a desk in a quiet room. The subject was instructed to place his or her left hand in a supinated position in a wooden box (450 mm × 900 mm × 100 mm), handmade for this study and placed on the desk. The subject’s left hand was hidden in the box. In place of the subject’s hand, a rubber hand (an adult cosmetic prosthesis) was placed in the same supinated position adjusted to the center of the subject’s sitting position approximately 15 cm to the right of the subject’s hand.

The experimenter stroked the subject’s palm and the palm of the rubber hand with two identical cosmetic brushes for 120 s, excluding the thumb, which was the test site of the subsequent tactile evaluation. The frequency of the brush stroking was set to 1 Hz and controlled by a metronome. This part of the experiment consisted of two conditions: the first in which the rubber hand and the subject’s hand were stroked simultaneously, wherein the illusion generally occurs (synchronous condition), and the second in which the rubber hand and the subject’s hand were stroked at different times, wherein the illusion generally does not occur (asynchronous condition). The order of the experimental conditions was counterbalanced among the subjects.

#### Questionnaire

To evaluate changes in the subjective sense of body ownership, subjects were asked to rate the following two statements, items modified from a questionnaire used by Lane et al. (2017).

Q1. “I feel as if the rubber hand is my hand.”

Q2. “I feel as if the real left hand is no longer mine.”

Question 1 queried the amount of illusory body ownership of the rubber hand (ownership score). Question 2 queried the amount of ownership decrease of the real hand due to the illusion (disownership score). Responses were collected verbally on a 7-point numerical rating scale and ranged from “−3” (very strongly disagree) to “+3” (very strongly agree). Pre-evaluation in the observation phase of the rubber hand before the synchronous or asynchronous stimulation was defined as the baseline to derive the amount of change of subjective evaluation, and to investigate the correlation with the amount of change of other indicators. Thus, a total of four measurements were conducted before and after the illusion-induced operation in the synchronous and asynchronous conditions.

#### Tactile Evaluation

**FIGURE 3:**
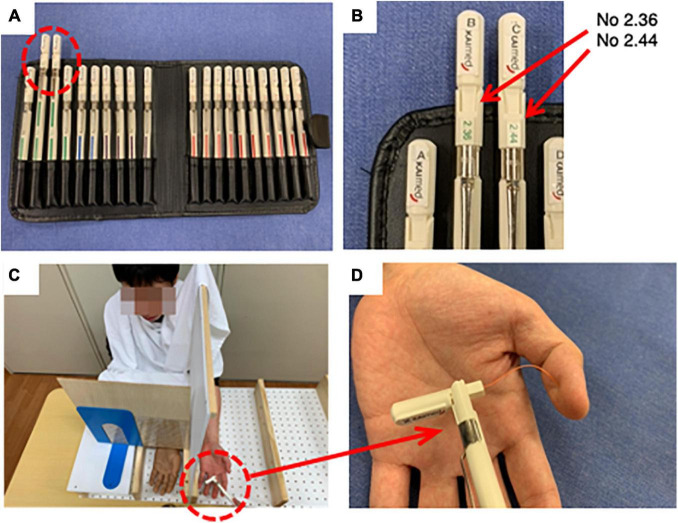
Settings of the tactile evaluation. **(A)** The Semmes-Weinstein monofilament (SWMT, SOT-DM20A, Sakai Medical Co., Ltd., Japan). **(B)** Two types of filaments used in this experiment, No. 2.36 (0.02 g) and No. 2.44 (0.04 g). **(C)** Settings of the tactile evaluation. **(D)** Detection site in tactile evaluation.

The tactile pressure evaluation was conducted immediately after the questionnaire on body ownership; the evaluation was conducted four times, as with the questionnaire measurements. A modified version of the method from Bell-Krotoski et al. (1993) was used for the evaluation. A Semmes-Weinstein monofilament (SWMT, SOT-DM20A, Sakai Medical Co., Ltd., Japan) was used for the tactile evaluation (see Figure 3A). Preliminary experiments with constant methods have shown that most healthy subjects have a sensory threshold between filaments No. 2.36 (0.02 g) and No. 2.44 (0.04 g). Thus, in this study those two filament types were selected from the kit (see Figure 3B). The experimenter held the monofilament lightly, lowered the tip of the filament vertically toward the subject’s thumb surface for 1–1.5 s, and released it for 1–1.5 s (see Figures 3C,D). The subjects were asked to respond “yes” when they felt pressure. The stimulation with each filament was performed 15 times, for a total of 30 instances. The order of stimuli and the order of interstimulus intervals were randomized.

### Statistical Analysis

The Shapiro–Wilk test was used to examine the distribution of all datasets. As the results of the questionnaire and of the tactile pressure sensation assessment were datasets that were not normally distributed, the Wilcoxon signed-rank test was used to analyze the difference between the means of the two datasets before and after the intervention. Spearman’s rank correlation coefficient was then used to examine the relationship between the amount of change in the decrease of body ownership score and the amount of change in the justification rate of tactile pressure sensation.

## Results

One of the participants who was recruited reported numbness in the arm during the experiment and ceased their participation. Therefore, data for 25 participants were obtained and analyzed.

### Ownership and Disownership Questionnaire Scores

**FIGURE 4:**
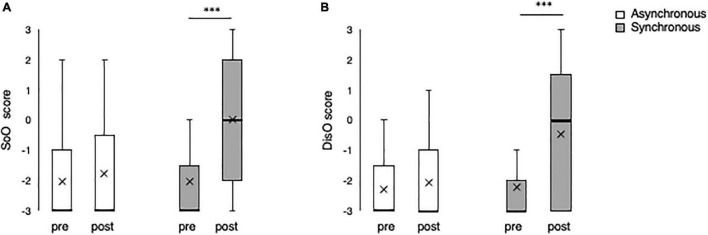
Ownership and disownership scores before and after the RHI. **(A)** Ownership score (SoO) from Question 1 in synchronous and asynchronous conditions. **(B)** Disownership scores (DisO) from Question 2 in synchronous and asynchronous conditions. Pre indicates results before the illusion operation, and post indicates results after the illusion operation. In the box-plots, the thick horizontal bars indicate the median, and the cross marks indicate the average values. ^***^p < 0.0005; error bars ± 1 SD.

The SoO scores (ownership score) from Q1 are shown in Figure 4A. In the synchronous condition, the median before the illusory manipulation was −3 (the interquartile range was between −3 and −2), and the median after the illusory manipulation was 0 (the interquartile range was between−3 and 1.5). In the asynchronous condition, the median before the illusory manipulation was −3 (the interquartile range was between −3 and −1.5), and the median after the illusory manipulation was −3 (the interquartile range was between −3 and −1). The ownership score for the synchronous condition changed significantly from pre-evaluation as the baseline (Wilcoxon signed-rank test, *p* < 0.0005), while in the asynchronous condition no significant change was revealed.

The decrease of ownership scores (disownership score) from Q2 are shown in Figure 4B. In the synchronous condition, the median before the illusory manipulation was −3 (the interquartile range was between −3 and −1.5), and the median after illusory manipulation was 0 (the interquartile range was between −2 and 2). In the asynchronous condition, the median before the illusory manipulation was −3 (the interquartile range was between −3 and −1), and the median after the illusory manipulation was −3 (the interquartile range was between −3 and −0.5). The disownership score for the synchronous condition changed significantly from pre-evaluation as the baseline (Wilcoxon signed-rank test, *p* < 0.0005), while in the asynchronous condition no significant change was revealed.

Both the ownership score and the disownership score was significantly higher in the synchronous condition after the illusory manipulation (post-sync) than in the asynchronous condition (post-async) (Wilcoxon signed-rank test, *p* < 0.0005). From these results, the RHI induction by the visuo-tactile stimulation has confirmed.

### Tactile Evaluation

**FIGURE 5:**
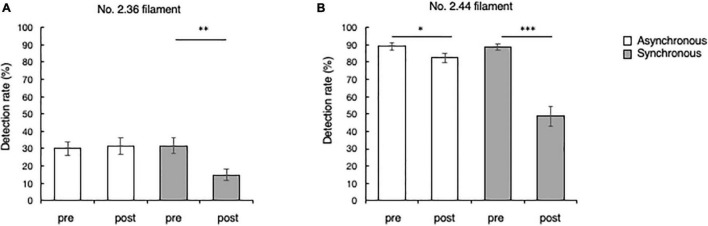
Tactile sensitivity before and after the RHI. **(A)** The detection rates of the No. 2.36 filament in the synchronous and asynchronous conditions. **(B)** The detection rates of the No. 2.44 filament in the synchronous and asynchronous conditions. Pre indicates results before the illusion operation, and post indicates results after the illusion operation. *p < 0.05; ^**^p < 0.005; ^***^p < 0.0005; error bars ± 1 SE.

The detection rate of the No. 2.36 filament in the tactile evaluation is shown in Figure 5A. No. 2.36 filament was thinner and harder to detect than the other one, so the detection rate was low overall both pre and post evaluations. The detection rate in the synchronous condition was 31.7 ± 4.5% before the illusion manipulation and 14.9 ± 3.5% after the illusion manipulation. While the detection rate in the asynchronous condition was 30.1 ± 4.0% before the illusion manipulation and 31.5 ± 4.7% after the illusion manipulation. In the synchronous condition, the detection rate of the No. 2.36 filament was significantly lower after the illusion operation than before the illusion operation (Wilcoxon signed-rank test, *p* = 0.002).

The detection rate of the No. 2.44 filament is shown in Figure 5B. The detection rate in the synchronous condition was 88.8 ± 1.6% before the illusion manipulation and 48.8 ± 5.5% after the illusion manipulation. The detection rate in the asynchronous condition was 89.1 ± 2.1% before the illusion manipulation and 82.4 ± 2.9% after the illusion manipulation. The detection rate of the No. 2.44 filament was significantly lower after the illusion operation than before the illusion operation under both synchronous (*p* < 0.0005) and asynchronous conditions (*p* = 0.030).

### Relationship Between the Amount of Change in the Body-Disownership Score and the Amount of Change in the Filament-Detection Rate

**FIGURE 6:**
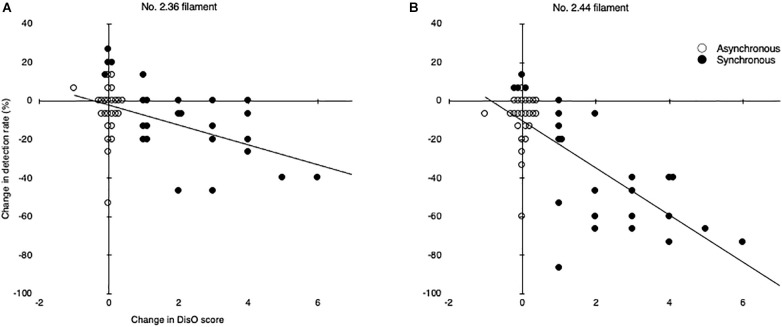
Scatterplots of disownership-score changes and detection-rate changes. **(A)** The relationship between disownership-score (DisO) changes and detection-rate changes for the No. 2.36 filament in all conditions. **(B)** The relationship between disownership-score (DisO) changes and detection-rate changes for the No. 2.44 filament in all conditions. Data from 25 subjects are plotted. Closed circles indicate plots under the synchronous condition. Open circles indicate plots under the asynchronous condition. Trend-lines show significant negative correlations for both No. 2.36 filaments (ρ = −0.478, *p* < 0.0005) and No. 2.44 filaments (ρ = −0.713, *p* < 0.0005).

To clarify the relationship between the decrease of the SoO and tactile detection ability, the correlation between subjective rating changes and detection-rate changes was investigated. The correlation between the amount of change in each score (ownership/disownership-score, detection-rate changes) is shown in Table 1. The subjective rating changes are indicated by subtracting the score measured before the illusion operation (pre-evaluation) from the score measured after the illusion operation (post-evaluation). The detection-rate changes are obtained by calculating the difference in the percentage of detection accuracy shown in Figure 5. Significant correlations were found between all pairs. A particularly important point was the correlations between the amount of change in the detection rate and the disownership score (see Figure 6). The amount of change in the detection rate of the No. 2.36 filament before and after the illusion showed a significant negative correlation with the amount of change in the disownership score (ρ = −0.478, *p* < 0.0005; Figure 6A). The amount of change in the detection rate of the No. 2.44 filament before and after the illusion also showed a particularly strong and significant negative correlation (ρ = −0.713, *p* < 0.0005; Figure 6B).

**TABLE 1:** Correlation coefficients between all the amount of changes.

	2	3	4
1. Change in SoO	0.857***	−0.657***	−0.719***
2. Change in DisO		−0.478***	−0.713***
3. Change in No. 2.36 detection			0.578***
4. Change in No. 2.44 detection			

***p < 0.0005, SoO, ownership score; DisO, disownership score. The values shown in bold are also mentioned in scatterplots (Figure 6).

## Discussion

In this study that synchronized visuo-tactile stimuli, subjects experienced feeling as if a rubber hand was part of their own body, while at the same time declaring a decrease of ownership toward their actual hand. There was a high correlation between the subjective ratings of Q1 and Q2. This correlation indicates deductively that embodiment of the rubber hand and disembodiment of the self-hand are complementary phenomena. In comparisons before and after presentation of the illusion stimulus, subjects’ filament-detection rates were significantly decreased after the illusion. Correlations were found between the disownership data obtained from the questionnaire and filament-detection rate changes; the greater the change in disownership, the greater the decrease in the filament-detection rate. These study results revealed that subjects’ tactile pressure thresholds increased when they felt a decrease of their sense of body ownership, induced by the illusion’s stimuli.

### Decreased Tactile Sensitivity Due to Feelings of Disownership Under the Illusion

Tactile information ascends primarily from the spinal thalamic tract and is detected when it reaches the primary somatosensory cortex (S1). From there, the information is further transmitted to higher association areas, such as the parietal and temporal lobes, resulting in more complex cognition, including renewal of body representation (Iwamura, 1998; Kandel et al., 2014). A previous study assessing evoked potential during the RHI showed a relative attenuation of somatosensory evoked responses in frontal electrodes corresponding to cortical sources in the higher sources of the parietal lobe in the attribution of ownership of an artificial hand (Zeller et al., 2015). This relative attenuation may reflect a decrease in the precision of somatosensory detection. Another SEP study on the full body illusion induced by applying visuo-tactile stimuli to other parts of the body demonstrated two distinct brain activity modulations. The activation around the time of the first parietal component of tibial nerve SEPs (P40) was enhanced during the illusion; the later activation, originating in higher somatosensory regions in the parietal cortex, had greater amplitude and longer duration in the non-illusion condition (Aspell et al., 2012). This study also suggested that the parietal lobe plays a significant role in the detection of visuo-tactile conflicts from each modality and modulating activity in the frontal network. These findings suggest the following three effects associated with disownership of one’s own body under the illusion, (1) multisensory integration effects (the sense of detecting tactile stimuli with the virtual body), (2) attentional modulation effects (the shift of spatial attention from the self-body to the virtual body) (Ortigue et al., 2006), and (3) functional deafferentation in the physical body. In the RHI stimulation in the present study, these three effects may have been induced in terms of the subjects’ hands. As a result, illusory ownership is likely to have made it difficult for the subjects’ actual hands to detect tactile stimuli under the illusion, and the detection rate for the tactile stimuli decreased.

Another recent psychophysical experiment has shown the opposite effect of suppressing tactile detection and promoting visual detection after the RHI (Rossi Sebastiano et al., 2021). This effect is considered to reflect the trade-off between downregulation of the somatosensory system during the RHI and increase of the connectivity between visual regions and the premotor cortex. Indeed, the functional connectivity to resolve the multisensory conflicts caused by RHI has been observed using neuroimaging (fMRI), electrophysiological (EEG) and near-infrared spectroscopy (NIRS) measurement data (Limanowski and Blankenburg, 2015; Arizono et al., 2016; Zeller et al., 2016). According to the predictive coding theory, in self-body identification by multisensory integration, the spatio-temporal mismatches between visual, tactile, and proprioceptive information associated with the bodily illusion produce a bottom-up effect on the parietal lobe, inducing a change in ownership. The updated information on integrated body representation in the parietal lobe provides a top-down inhibition of prediction errors on sensory processing in the frontoparietal network (Tsakiris and Haggard, 2005; Costantini and Haggard, 2007; Apps and Tsakiris, 2014). It is surmised that the visuo-tactile stimuli in the present experiment provided a top-down inhibitory effect on sensory processing similar to the effect in previous studies, and that this inhibition was one of the causes of decreased sensory detection.

### Relationship Between the Tactile Sensitivity and Intensity of Illusory Ownership

In the tactile-pressure evaluation test conducted before and after the visuo-tactile stimulation in this study, the filament-detection rate was significantly lower after stimulation than before stimulation under all conditions (except for the detection of the thin filament, No. 2.36, under asynchronous conditions due to the poor detection rate both before and after stimulation). In addition, negative correlations were observed between the amount of change in the filament-detection rate and the change in the disownership score, collected *via* the questionnaire, in all conditions regardless of the filament applied (No. 2.36 or No. 2.44). These correlations indicate that the tactile-pressure threshold increased relatively as feelings of ownership of their real hand decreased significantly because of the RHI, resulting in difficulty detecting the tactile stimuli.

In a previous study investigating changes in tactile-detection performance after the presentation of illusion stimuli, the detection of vibration after synchronous conditions was significantly lower than detection after asynchronous conditions (Zopf et al., 2011). Although this study found that observing one’s own body is a major reason for decreased tactile detection, the study did not consider the standalone effect of changes in body ownership, excluding visual information. The present evaluation shielded the contact area during the clinical examination and removed the suppressing effect of the detection of tactile stimuli by visual information. Note that the detection threshold was specified in more detail by measuring the tactile-detection rate before and after offering the illusion stimuli using two types of filaments. The disownership caused by the illusion-inducing stimuli attenuated the accuracy of tactile detection even without observing the corresponding body parts. This is a novel finding that differs from findings of previous studies.

Physiological changes induced by illusory body ownership include less activation of motor-evoked potential of the corresponding body part (Della Gatta et al., 2016), an attenuation in SEPs for subsequent sensory input (Zeller et al., 2015), modulation of tactile and visual detectability (Zopf et al., 2011; Rossi Sebastiano et al., 2021), and delays in temporal order judgments with decreased skin temperature (Moseley et al., 2008; Salomon et al., 2013). All of this evidence was obtained by comparing illusion and non-illusion conditions. The evidence did not explain the relationship between the intensity of the illusion and the physiological changes; the relationship between the susceptibility to illusion and the intensity of illusory body ownership also remains unclear. Thus, subjective evaluation using the conventional questionnaire can detect the occurrence of illusions but cannot make a quantitative evaluation of the intensity of illusory ownership. Study results show that there were significant changes in the sensory detection rate and negative correlations with the disownership changes, indicating the existence of subtle changes in the SoO that were indiscernible using the 7-point scaled questionnaire and conventional methodology. The amount of change in the filament-detection rate, a quantitative index of physiological evaluation, reflected changes in the disownership of subjects’ own bodies according to the intensity of the illusion.

In the study’s experimental environment, the rubber hand was placed next to the subject’s own hand in a homologous orientation, which is generally prone to be under the illusion. Therefore, most subjects reported a change in ownership through the illusion manipulation of their non-dominant hands. The detection rate of thick (No. 2.44) filaments was significantly reduced in the study, even under asynchronous conditions. A previous study reported that visual information from a first-person perspective enabled the provision of illusory ownership even under asynchronous visuo-tactile stimulation (Maselli and Slater, 2013). The current study’s results indicate that the asynchronous visuo-tactile stimuli also induced illusory ownership, though not as much as under the synchronous conditions. The evaluation of tactile sensitivity in this study also revealed a slight change in illusory ownership under asynchronous conditions.

### Clinical Applications

The present study demonstrated that illusion-induced stimulation in healthy adults leads to a decrease in sensory detection as well as to a decrease in the SoO of the subjects’ own hands. In clinical cases, many stroke patients declaring disownership showed severe sensory and motor deficits (Ronchi et al., 2020), indicating that the reduced frequency of use for the affected limb, as well as the brain damage in areas involved in sensory and motor function, might be the causes of the decrease of ownership. This study’s experimentally generated situation in illusory ownership may be useful in understanding the pathological condition of patients with sensory and motor deficits.

A previous study using RHI with healthy subjects reported that the illusion had a greater effect on the non-dominant than on the dominant hand (Dempsey-Jones and Kritikos, 2019). Another study that included a group of healthy subjects who prevented left-hand movements by using a cast for 1 week also showed stronger illusory effects on the immobilized hand and weaker illusory effects on the non-immobilized hand (Burin et al., 2017). Expansion of the subject group of hemiplegic patients also showed that patients displayed stronger illusory effects on their paretic hand than the intact hand (Burin et al., 2015). These findings indicate that body parts used infrequently in daily life are generally vulnerable to manipulation in body ownership. Specifically, hemiplegic patients with somatosensory disorders have relatively decreased use frequency of their paretic limbs in daily life, leading those patients to experience further decrease of the SoO of their paretic limbs and, thus, leading to further nonuse.

Results of the present study revealed that the sense of disownership induced by the RHI is an inhibiting factor for sensory detection. Based on this finding, optimizing the SoO of limbs with sensory impairment and decreasing the occurrence of learned nonuse may contribute to the normalization of sensory function. These trials are expected to lead to innovations in rehabilitation strategies.

### Limitations

First, the ownership and disownership scores in this study were obtained from questionnaires completed by healthy subjects only; similar results describing changes in the SoO due to illness or trauma are not guaranteed. The causal relationship between the loss of the sense of body ownership and sensory dysfunction in clinical cases remains unclear. Future studies will require the same questionnaire evaluations from clinical patients. Second, the tactile pressure evaluation’s examination region was limited to part of the left thumb; therefore, changes in sensory function throughout the entire hand or in other body parts are unknown. Future investigations that examine if the same physiological change extends to the whole body are necessary and should utilize other experimental set-ups, such as full body illusion.

## Conclusion

A sense of disownership invoked by inducing the RHI in healthy subjects led to an increase in those subjects’ tactile-pressure thresholds. This result suggests that decrease of body ownership is an important factor in changing the function of tactile sensation. Interventions that alter the sense of body ownership may contribute to the rehabilitation of sensory impairment after stroke.

## Data Availability Statement

The raw data supporting the conclusions of this article will be made available by the authors, without undue reservation.

## Ethics Statement

The studies involving human participants were reviewed and approved by the Ethics Committee of Tohoku University School of Medicine (registration number: 2020-1-47). The patients/participants provided their written informed consent to participate in this study.

## Author Contributions

KA, TS, and S-II conceived and designed the experiment. KA developed the experimental system, conducted the experiment, and statistical analysis. TS, RO, and ES provided critical review of the experimental procedure and data analysis. KA and TS wrote the draft of the manuscript. All authors contributed to the discussion of the results and approved the final manuscript.

## Conflict of Interest

The authors declare that the research was conducted in the absence of any commercial or financial relationships that could be construed as a potential conflict of interest.

## Publisher’s Note

All claims expressed in this article are solely those of the authors and do not necessarily represent those of their affiliated organizations, or those of the publisher, the editors and the reviewers. Any product that may be evaluated in this article, or claim that may be made by its manufacturer, is not guaranteed or endorsed by the publisher.
